# Graphene Oxide Scaffold Stimulates Differentiation and Proangiogenic Activities of Myogenic Progenitor Cells

**DOI:** 10.3390/ijms21114173

**Published:** 2020-06-11

**Authors:** Mateusz Wierzbicki, Anna Hotowy, Marta Kutwin, Sławomir Jaworski, Jaśmina Bałaban, Malwina Sosnowska, Barbara Wójcik, Aleksandra Wędzińska, André Chwalibog, Ewa Sawosz

**Affiliations:** 1 Department of Nanobiotechnology and Experimental Ecology, Institute of Biology, Warsaw University of Life Sciences, Ciszewskiego 8, 02-786 Warsaw, Poland; anna_hotowy@sggw.edu.pl (A.H.); marta_kutwin@sggw.edu.pl (M.K.); slawomir_jaworski@sggw.edu.pl (S.J.); jasmina_balaban@sggw.edu.pl (J.B.); malwina_sosnowska@sggw.edu.pl (M.S.); barbara_wojcik@sggw.edu.pl (B.W.); a.wedzinska1@gmail.com (A.W.); ewa_sawosz_chwalibog@sggw.edu.pl (E.S.); 2 Department of Veterinary and Animal Sciences, University of Copenhagen, Groennegaardsvej 3, 1870 Frederiksberg, Denmark; ach@sund.ku.dk

**Keywords:** angiogenesis, myogenic progenitor cells, graphene oxide

## Abstract

The physiological process of muscle regeneration is quite limited due to low satellite cell quantity and also the inability to regenerate and reconstruct niche tissue. The purpose of the study was to examine whether a graphene oxide scaffold is able to stimulate myogenic progenitor cell proliferation and the endocrine functions of differentiating cells, and therefore, their active participation in the construction of muscle tissue. Studies were carried out using mesenchymal cells taken from 6-day-old chicken embryos and human umbilical vein endothelial cells (HUVEC) were used to assess angiogenesis. The graphene scaffold was readily colonized by myogenic progenitor cells and the cells dissected from heart, brain, eye, and blood vessels did not avoid the scaffold. The scaffold strongly induced myogenic progenitor cell signaling pathways and simultaneously activated proangiogenic signaling pathways via exocrine vascular endothelial growth factor (VEGF) secretion. The present study revealed that the graphene oxide (GO) scaffold initiates the processes of muscle cell differentiation due to mechanical interaction with myogenic progenitor cell.

## 1. Introduction

Degradation, degeneration, and surgical removal are among the most common causes of muscle loss. These processes are largely irreversible. Despite the ability of muscle cells to regenerate, the physiological process of regeneration is quite limited. This is because there exist a limited number of satellite cells, and also a result of the inability of a muscle to regenerate and reconstruct its niche. The niche, which is the immediate environment of stem cells, well cell availability and growth factors mutually influence each other and are required for the regeneration of tissues [1]. However, the interactions between these factors are not fully understood. It seems that designing an appropriate niche—one that is optimally suited for promoting cellular regeneration—is the most difficult challenge faced by researchers who aim to enhance the regeneration of muscle tissue [2]. The creation of a new tissue, however, involves interactions between three basic elements: cells capable of proliferation, differentiation, and creating interconnections; scaffolds that act as mechanical foundations for growth; and growth and functional factors [3]. Many studies have been devoted to the invention and adaptation of synthetic or biological niche environments for tissue engineering.

Carbon is relatively biocompatible and is a promising scaffold material. Carbon nanomaterials can be used to create scaffolds of various structural types and can impart features of regularity and, most importantly, flexibility. This allows for the imitation of the physiological structures characteristic of the muscle niche. Graphene, particularly, graphene oxide (GO), easily attaches to other compounds through interactions involving the surface oxygen groups and can create a surface containing a mosaic of chemical groups that are exposed to the environment [4]. It has a great physical and chemical plasticity and possesses a strong and durable graphene mesh structure [5,6], which possesses favorable characteristics and facilitates formation of a scaffold with the potential action as a graphene-derived synthetic extracellular matrix (ECM) structure [7]. The presence of numerous oxygen groups on the GO surface enhances its hydrophilicity and biocompatibility [8], improving its biological applicability.

Key elements of tissue formation involve interactions between the ECM and the cells that are capable of proliferation, differentiation, and creation of interconnections. However, many key phenomena involved in these interactions have not been sufficiently elucidated. In particular, the physicochemical impact of components of the ECM on myogenic progenitor cell proliferation, differentiation, tissue formation, and, above all, activation of extracellular signaling are not clear. The embryonic chicken muscle development model can provide a precise, convenient and rapid means to study interactions between the myogenic progenitor cells and the potential ECM mimics. Muscle tissues of chicken embryos are formed similarly to those of other animals. Multipotent stem cells of the mesoderm ignite somites and leg buds arise from somites 26 to 32 throughout 17 Hamburger–Hamilton stages (HH) (52–64 h) and up to 26 HH (day 5) [9]. Stimulated myoblast precursors migrate to the limb bud and are guided and nourished by the substances of the extracellular matrix present in the peripheral mesenchyme of the limb bud.

Proliferating precursors of muscle cells have express specific proteins throughout specific stages of tissue development, which extends from proliferation to differentiation. In order from the earliest to the latest, the proteins expressed include MYF5 (myogenic factor 5), MyoD (myogenic differentiation 1), MRF4 (myogenic factor 6), and myogenin. MyoD controls the chromatin context and manages the genes involved in myogenic programming [10]. MyoD is a marker of skeletal muscle cells and, together with Myf5, is essential for the establishment of a viable muscle lineage [11].

Muscle cells and their precursors, however, exhibit not only endocrine, but also paracrine properties. In vitro studies of muscle satellite cells co-cultured with endothelial cells have found them to greatly stimulate angiogenesis [12]. Moreover, VEGF secretion by muscle satellite cells was observed, indicating that muscle satellite cells secrete proangiogenic factors into the environment and create a niche for their development and the formation of muscle tissue. Another study showed that the coupling between the ECM and myogenic precursor cells (MPCs) activated myogenesis and angiogenesis [13]. Cooperation between muscle and endothelial cell regeneration at the molecular level (production of apelin, periostin, and oncostatin) conditioned the regeneration of muscle.

The purpose of the present study was to examine whether GO scaffolds are able to stimulate the myogenic progenitor cell differentiation, which includes the stimulation of endocrine properties and enhances their active participation in the construction of muscle tissue. The research is focused on the scaffold interaction with the cells and its role in the promotion of tissue formation.

## 2. Results

### 2.1. GO Scaffold Characterisation

**Figure 1:**
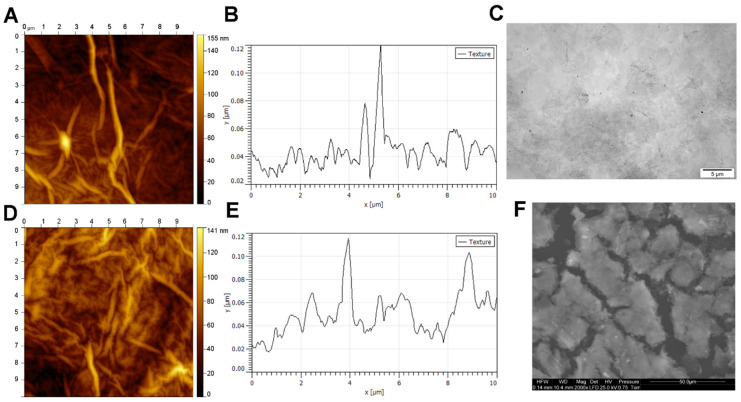
Graphene oxide scaffold morphology. (**A**) Atomic force microscopy images and (**B**) a topography model of the surface of the graphene oxide scaffold on a polystyrene culture plate. (**C**) Transmission electron microscopy image of graphene oxide. (**D**) Atomic force microscopy images and (**E**) a topography model of the graphene oxide scaffold surface on a flat silicon wafer. (**F**) Scanning electron microscopy image of the graphene oxide scaffold.

GO (50 mg/L) formed a stable aqueous hydrocolloid (zeta potential: −27.0 mV) that showed GO flakes with an average diameter from 5 to 30 μm via transmission electron microscopy (TEM) (Figure 1C). The GO scaffold was analyzed using scanning electron microscopy (SEM) and atomic force microscopy (AFM). A 1 mg/mL aqueous GO hydrocolloid was used to prepare the GO scaffold. SEM images showed layers of GO flakes covering the surface of cell culture plates (Figure 1F). AFM analysis was performed to assess the GO scaffold on a cell culture plate and a perfectly flat silicon wafer. AFM analysis of the GO scaffold on the silicon wafer showed that GO formed a surface with sharp peaks and the average roughness of 5.1 nm (Figure 1D,E). The GO scaffold created using a polystyrene cell culture surface had an increased number of peaks but a reduced average roughness of 2.0 nm (Figure 1A,B), which indicated that GO fills cavities of polystyrene cell cultures, which leaves surfaces less rough.

### 2.2. PMC Localise Preferentially on GO Scaffold

**Figure 2:**
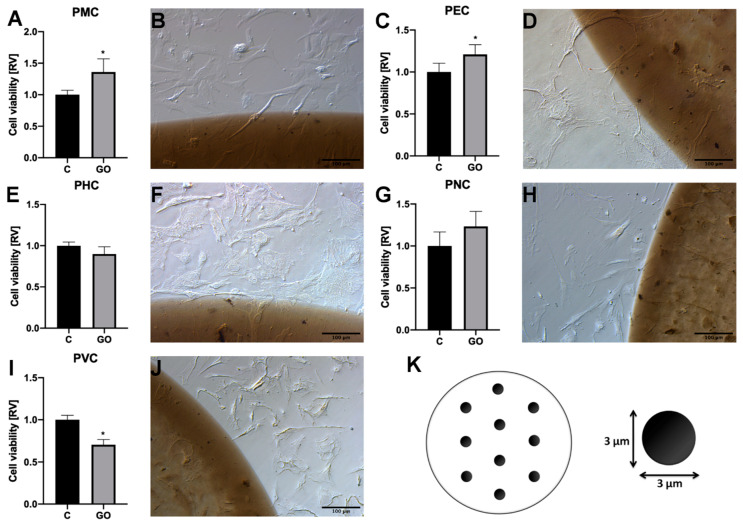
Analysis of the cell localization and viability on the GO scaffold. (**A**) Viability and (**B**) morphologies of progenitor muscle cells from hind limb (PMC) on the GO scaffold. (**C**) Viability and (**D**) morphology of progenitor eye cells (PEC) on the GO scaffold. (**E**) Viability and (**F**) morphology of progenitor heart-derived cells (PHC) on the GO scaffold. (**G**) Viability and (**H**) morphology of cells derived from brain (progenitor nerve cells, PNC) on the GO scaffold. (**I**) Viability and (**J**) morphology of cells from a chorioallantoic membrane’s blood vessel (progenitor vessel cells, PVC) on the GO scaffold. Morphology was assessed on the edge of the GO scaffold via light microscopy with phase contrast and 200 × magnification. Statistical significance is indicated with different superscripts (unpaired *t*-test; *p* < 0.05). (**K**) Pattern of GO islands used in cell localization and morphology analysis. Acronyms: C, control; GO, graphene oxide; RV, relative value.

Organs/tissues dissected from a different part of the body (a hind limb (PMC), heart (PHC), brain (PNC), an eye (PEC), and a blood vessel from the chorioallantoic membrane (PVC)) were sources of mesenchymal cells involved in development of specific organs or tissues. GO enhanced viability of PMC and PEC, but decreased viability of PVC and did not affect PNC and PHC viability (Figure 2). To clearly observe morphological differences between the cells grown on the GO scaffold and polystyrene, as well as to assess the direction of movement and homing preference of the GO surface, an island pattern was used (Figure 2K). Thus, we compared morphologies of cells on the GO island with those growing beyond GO and on the border of the GO island. A morphological assessment of cells revealed that cells were typical for the period of embryonic development assessed. Mesenchymal stem cells and differentiated cells were observed in each sample. All cells were located on the polystyrene surface, on the border of the GO island, and on the GO island, however, they varied with respect to density and morphology (Figure 2). PMC, unlike other observed cells, actively localized on GO islands. (Figure 2B), whereas non-PMC were located outside the GO. Images of the GO island border showed that cells predominantly localized parallel to the GO border, however, sometimes they attached to the GO scaffold (Figure 2D,F,H,J). The GO scaffold was not definitively avoided by PHC, PNC, PEC, and PVC, however, any preference to locate on the GO was not observed.

**Figure 3:**
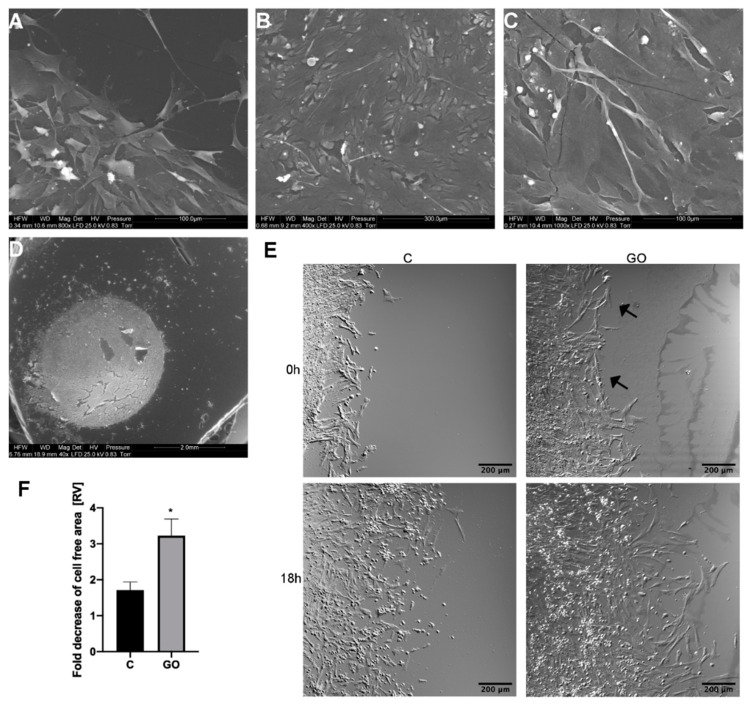
PMC morphology after a 96-h incubation and PMC migration on a GO scaffold. (**A**) Scanning electron microscope visualization of PMC on the edge and (**B**,**C**) the center of the GO scaffold. (**D**) Visualization of the whole GO scaffold overgrown by PMC. (E) Representative images of the migration analysis of PMC throughout the GO scaffold. Image of the initial (“0 h”) position of the PMC and their positions after an 18-h incubation period on a polystyrene culture plate (“C”) and a graphene oxide scaffold (“GO”). (F) Fold decreases of the cell-free areas measured as the difference determined between 0 h and 18 h images. Arrows indicate the edge of the GO scaffold. Statistical significance is indicated with different superscripts (unpaired *t*-test; *p* < 0.05). Abbreviations: C, control; GO, graphene oxide; RV, relative value.

To verify PMC behavior on the GO scaffold, SEM visualization was carried out after the cells were incubated with the GO scaffold for extended periods. PMC incubated for 96 h and imaged via SEM showed a tendency to localize on GO islands. At the border of GO islands, cells were placed perpendicularly to the GO circle with protrusions directed towards the GO surface (Figure 3A). SEM imaging allowed researchers to distinguish between two layers of PMC. The first one was large and contained flattened cells located at the bottom of wells. These cells adhered to the GO scaffold. The second layer of cells grew over the bottom cell layer and consisted of oval, slightly rectangular single and fused myoblasts (Figure 3B,C). Cells of the second layer, myogenic progenitor cells and differentiated myocytes, had cytoplasmic protrusions that were connected to cells of the first layer.

### 2.3. The GO Scaffold Increases Migration of PMC

To assess PMC surface preference, cell migration speed on the control polystyrene and the polystyrene covered with a GO scaffold were analyzed. Analysis of migration was initiated when the cell reached the edge of the scaffold and began to migrate to the GO scaffold. Cells in the control group (Figure 3E, “C”) covered the polystyrene surface during the 18-h time-lapse imaging period almost two times slower than the cells that migrated next to the GO scaffold (Figure 3E, “GO”).

### 2.4. The GO Scaffold Increases Expression of the MyoD1 mRNA and Enhances Synthesis of VEGF-A Proteins

**Figure 4:**
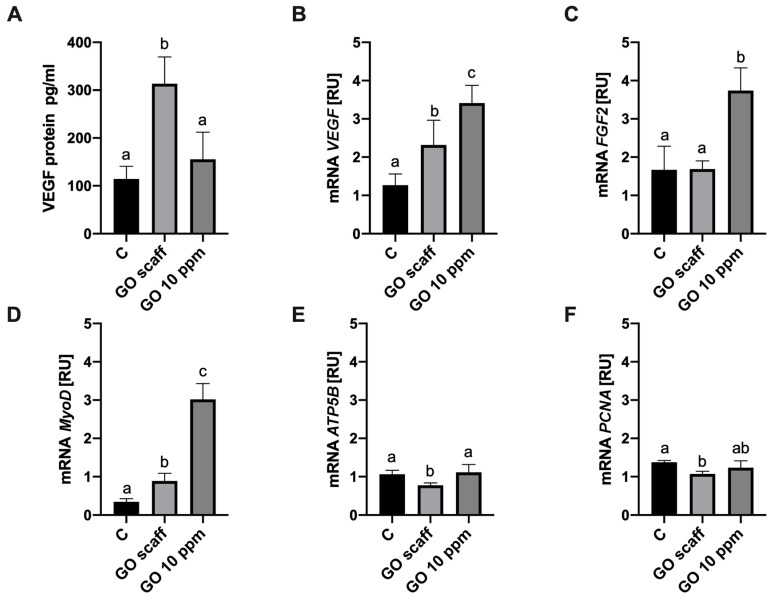
Analysis of mRNA expression and protein level. (**A**) Vascular endothelial growth factor A (VEGF-A) protein level in the cell medium after incubation of PMC on a standard cell culture plate (control—“C”), PMC on the graphene oxide scaffold (“GO scaff”), PMC on a standard cell culture plate treated with GO (final concentration—10 μg/mL). Expression level of the genes coding (**B**) VEGF-A, (**C**) basic fibroblast growth factor (bFGF), (**D**) myoblast determination protein 1 MyoD, (**E**) ATP synthase F1 subunit beta ATP5B, (**F**) proliferating cell nuclear antigen PCNA. Statistical significance is indicated with different superscripts (one-way ANOVA; *p* < 0.05). Abbreviations: RU, relative units.

To explain mechanism of the PMC–GO scaffold interaction, mRNA expression levels of the key genes responsible for myogenic progenitor cell differentiation, proliferation, and maturation were assessed. The GO scaffold influenced expression of the genes involved in myogenic progenitor cell proliferation and differentiation. The GO surface enhanced expression of MyoD1, which is the gene responsible for differentiation, and decreased expression of the proliferating cell nuclear antigen (PCNA) involved in proliferation and of ATP5B (coding ATP synthase F1 subunit beta). However, the GO scaffold did not affect basic fibroblast growth factor (bFGF expression (Figure 4). Interestingly, VEGF-A gene indicating the angiogenesis process was upregulated after application of the GO scaffold. To analyze cell secretion of VEGF-A, the level of this protein in media was assessed. The concentration of VEGF-A in the culture medium from the PMC incubated on the GO surface was increased as compared to the control (Figure 4).

**Figure 5:**
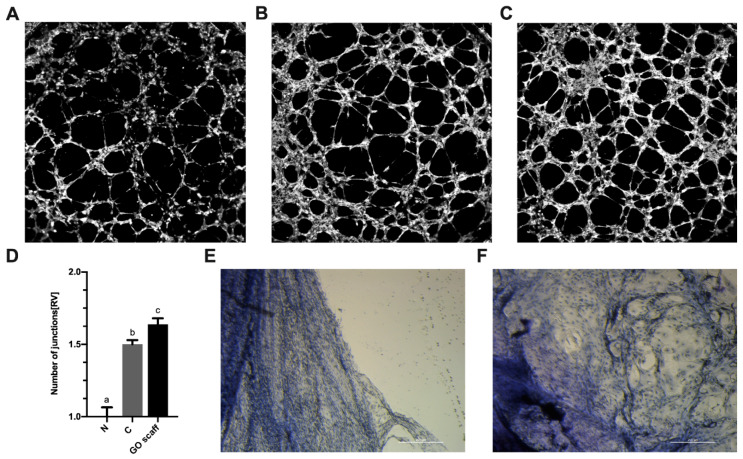
Analysis of angiogenic properties of PMC media. Images of human umbilical vein endothelial cells (HUVEC) tube formation in (**A**) the control medium and (**B**) the negative control medium with the post-incubation addition of the medium from the PMC cultured under standard conditions as the control, and (**C**) with the post-incubation addition of the medium from the PMC cultured on a graphene oxide scaffold. (**D**) Graph showing the mean number of junctions of HUVEC tubes in the field of view. Values are expressed as mean ± standard deviation. Statistical significance is indicated with different superscripts (one-way ANOVA; *p* < 0.05). (**E**) Implant from the peripheral blood vessel of a chicken embryo incubated in the medium derived from standard PMC cultures (negative control) and (**F**) analysis of cells incubated in the medium from the PMC cultured on graphene oxide. Abbreviations: RV, relative value; N, negative control; C, control; GO scaff, experimental group.

To confirm the release of VEGF-A, PMC cultured on a GO surface ex ovo and tube formation angiogenesis models were assessed. To evaluate proangiogenic properties of the PMC culture media, a GO scaffold human umbilical vein endothelial cells (HUVEC) tube formation assay was performed. The medium from the PMC cultured on a GO scaffold was used as an experimental group (Figure 5C), the medium from the PMC cultured on a standard polystyrene surface was used as a control (Figure 5B). Additionally, freshly supplemented DMEM was used as a negative control (Figure 5A). The number of tube junctions formed after incubation with experimental and control media indicated angiogenic potential. The number of junctions formed after exposure to the experimental group was statistically higher than that of the control and negative control groups (Figure 5D).

Additionally, proangiogenic properties of the medium were verified using an implant taken from the peripheral blood vessel of a chicken embryo. After a 3-d incubation period, the vascular implants subjected to culture media from the control PMC and the PMC cultivated on a GO scaffold significantly differed. The fragment of the vessel that was cultured in the presence of the control PMC media was weakly altered. Groups of single cells in the vicinity of dense vessel tissue were clearly visible (Figure 5E,F). Morphological features of these cells corresponded to those of endothelial cells. A blood vessel scrap grown in the presence of the medium from the PMC cultured on a GO scaffold was more drastically altered. Its edges were more jagged, and cells migrated away from the sample.

## 3. Discussion

GO is an extremely thin, resilient, light, and durable structure. It is considered a basement, or ECM-equivalent, structure. According to Kenry et al. [14], superior properties of graphene and graphene-like materials may enhance the regeneration capacity of tissues in a controlled way. However, application of GO as a substructure for cellular proliferation and differentiation requires examination of its toxicity. The problem of potential GO toxicity has inspired an increasing number of in vitro and in vivo studies. Several studies showed that intratracheal exposure to GO is highly toxic [15]. Sydlik et al. [16] stated that the GO implanted subcutaneously and intraperitoneally was moderately compatible with the inflammatory response. In the previous work, we showed that the short- and long-term exposure of rats to intraperitoneal GO did not produce toxicity [17,18]. It seems that toxicity of GO remains controversial and depends on many factors, including the graphene source, the biological medium used, and the experimental protocol. When introduced to the culture medium, GO was internalized and localized to F-actin filaments, which altered cell–cycle progression, apoptosis, and oxidative stress [19]. In our experiments, however, GO was immobilized as a scaffold and did not interact with the actin inside the cell. In vitro experiments carried out using standard methods introduced experimental nanomaterials to the culture medium, but insufficiently evaluated affinity. For this reason, an “affinity test” was performed. All cells were seeded on 6-well plates using an island pattern, which was prepared using the GO hydrocolloid. This allowed researchers to observe behavior of the cells that were free to undergo both migration and colonization of GO islands or plate areas between islands. This “nano-island” strategy was used to assess protein-detecting sensors [20] by Lunova et al. [21], who assessed properties of liver cancer cells. In our experiments, we used a similar island method to assess cell affinity and propensity for colonization.

Another group of studies have determined that different levels of toxicity for GO depend on the chemical groups available on the surface of the material, and primarily on the amount of oxygen, especially of the C = O groups, available, which is inversely proportional to toxicity [22,23]. In this study, GO with a moderately high oxygen content (39–49%) was evaluated. Differences in GO biocompatibility also depend on biological models and the type of cells used. We have shown that expected differences in biocompatibility of GO can be observed using cells taken from different areas of the embryo. GO was investigated as a nanoscaffold prepared by applying and drying GO flakes that had been suspended in water at the bottom of a culture plate. Self-assembly of GO flakes produced a very thin layer that adhered to the GO scaffold at the bottom of the dish. The cells dissected from the peripheral blood vessels (PVC) reacted negatively to the GO scaffold. In contrast, the cells dissected from muscle (PMC) and eye (PEC) tissue had increased numbers when exposed to GO and GO did not affect growth of heart (PHC)- and brain (PNC)-derived cells. Thus, it can be assumed that in regenerative medicine, consideration must be paid to using compatible cells and surfaces.

Here, we evaluate the potential of GO to act as the ECM. The nanoscaffold’s ECM-like surface should be non-toxic and provide an adequate surface for cell adherence. Interestingly, all types of cells were observed attached to the GO scaffold. Regardless of the cell type, graphene islands were never completely avoided by cells. This indicated that GO was moderately biocompatible and confirmed the previous experiments that used GO as a nanoscaffold component. Introducing GO to polycaprolactone nanofiber scaffolds increased differentiation of mouse marrow mesenchymal stem cells (mMSC) to osteo-like cells and rat pheochromocytoma cells (PC12-L) to neuro-like cells [24]. Moreover, a chicken embryo muscle extract together with GO were used for the maturation of muscle progenitor cells [25]. Biocompatible GO was also used to reinforce gelatin–hydrphyapatitie 3D scaffolds and promoted bone regeneration in orthopedics [26]. Moreover, GO was used to reinforce electrospun gelatin or PCL scaffolds, increased tensile strength, and provided energy at the site of a break [27]. In other studies, GO–PCL and GO sheets were shown to be biocompatible. Further, they both facilitated myoblast differentiation from human mesenchymal stem cells [28]. The authors suggested that GO sheets and GO-based scaffolds were the most favorable hydrocolloids assessed for promoting skeletal muscle and other tissue regeneration. Additionally, other authors indicated that the reduced graphene oxide nanoribbon grid facilitated rapid osteogenic differentiation [29].

In light of the results of our experiments assessing different types of mesenchymal cells, GO was most suitable for regenerating muscle cells. Effects of extended incubation on migration revealed a strong affinity of PMC for the GO scaffold. This may be a result of mechanical or/and chemical properties of the GO scaffold surface. Mechanical properties, particularly with respect to elastic modulus, of mammalian cells (between 1 to 100 kPa [30]) vary. Mechanotransduction-sensitive signaling regulates myogenic differentiation, as well as endocrine and exocrine activities of these cells. It has been demonstrated that elasticity (12 kPa) of the matrix of myogenic progenitor cells influences their capacity to self-renew and restore muscle tissue [31]. In other studies, polyurethane–nano-GO nanofibrous scaffold was used for stimulation of myogenic differentiation due to nanofibrous morphology and high mechanical flexibility [32].

The challenge of creating nanosurface involving cellular interactions was discussed by Liu and Tang [33], who argued that ECM-like nanoparticle structures influence cellular movement and attachment, which can be visualized via assessment of lamellipodia or/and filopodia occurrence. In our experiment, the filopodia of all cells were observed at the borders of scaffold islands, mainly in the cells located parallel to GO islands, and filopodia were directed towards the scaffold. PMC, however, had lamellipodia that were positioned outstretched on the GO scaffold surface perpendicular to the GO surface. Visualization of PMC with SEM revealed the tendency of cells to migrate, attach to GO scaffolds, and revealed their preference to settle on GO islands. Moreover, the specific organization of cells throughout growth was observed. Tissues collected from embryonic hind buds consisted of undifferentiated cells, which differentiated and fused to form multinucleated myofibers. During embryogenesis, primary myotubes are established on day 4–7 to form the scaffold and to differentiate to form secondary myotubes on day 8–16 [34]. In our experiments, visualization via SEM revealed scaffolding layers that were created by primary muscle cells. Further, differentiating elongate and thin cells were connected to scaffolding cells. However, muscle fibers only formed when cells were connected to undifferentiated scaffolding cells to form a feeding-like layer or if they were connected to GO. Moreover, GO strongly promoted the signaling required for differentiation. An assessment of MyoD gene expression in the PMC incubated on either GO scaffolds or in the presence of the GO hydrocolloids revealed increased MyoD expression. MyoD is an early determinant of myogenic progenitor cells to the myogenic lineage. In particular, MyoD stimulates myoblasts to promote differentiation [35]. This regulator of myogenesis is expressed in proliferating myoblasts [36] and is capable of transdifferentiating many different kinds of cells to muscle cells [10,37]. Our results revealing increased expression of MyoD in the cells confirm the hypothesis that GO has pro-differentiation, rather than pro-proliferative, effects. Moreover, myoblast differentiation is correlated with decreased expression of a regulator of proliferation, PCNA, a phenomenon that was also observed in this study. However, expression of MyoD genes is often negatively correlated with bFGF expression [38]. In our study, exposure to a scaffold was not accompanied with the decreased expression of FGF2.

Studies of scaffolds also emphasized the role of the environment in shaping the regeneration of muscle tissue, especially hypoxia [31,39]. During ageing and muscle degeneration, satellite cells lose their ability to communicate and interact with the cells involved in angiogenesis [40]. The formation of blood vessels is fundamental for formatting new tissue, because they facilitate the delivery of oxygen, nutrients, and functional factors to cells. Therefore, the induction of angiogenesis by scaffolds enhances tissue regeneration. Acellular collagen–heparin scaffolds showed induced angiogenesis by secretion of VEGF and FGF2, ensuring lack of hypoxic cells [41]. In different studies, release of VEGF and IGF-1 from macroporous scaffolds enhanced muscle regeneration [42]. In our investigation, angiogenesis was induced due to upregulation of VEGF-A transcription and protein synthesis after PMC were cultured on a GO scaffold. In experiments using a mouse multipotent mesenchymal progenitor muscle cell line, C2C12, Bryan et al. [43] showed that VEGF expression is coordinately controlled with myogenic differentiation. Moreover, the study showed that MyoD is necessary for maintenance of proper levels of VEGF expression and endogenous synthesis of MyoD promotes VEGF expression. These findings are in accordance with our results, which showed that increased expression of MyoD is accompanied by increased VEGF levels, however, we also showed that increasing VEGF expression at transcript and protein levels compared with controls occurred in the cells cultured with GO scaffolds. In other experiments, activation of VEGF expression in mesenchymal stromal cells after vibration stimulation was observed [27]. Moreover, multi-layered electrospun scaffolds with macroporosity were used to facilitate angiogenesis in a tissue-engineered smooth muscle. The 25% macroporous group showed increased angiogenesis and improved implanted cell survival [44]. Mechanical impact on the cell likely triggers mechanisms determining cell fate. The GO scaffold induced VEGF release by myogenic cells, which indicated that surface and cell interactions play a key role in differentiation. Myogenic progenitor cells used in our experiments exhibited a preference for the GO scaffold and subsequently initiated differentiation.

This study revealed that the GO scaffold was readily populated and colonized by PMC and simultaneously activated exocrine VEGF secretion. Our findings show that GO scaffolds can initiate processes of muscle cell differentiation and stimulate angiogenesis due to mechanical interaction with PMC.

## 4. Materials and Methods

### 4.1. Graphene Oxide Scaffold

A GO water suspension (4 mg/mL) was purchased from Nanopoz, Poznań. GO flakes were produced from graphite using a modified Hummers method. Experimental scaffolds comprised of GO were prepared as previously described [45]. In short, the GO hydrocolloid (1 mg/mL) was applied to the bottom of a 6-well plate and dried in a laminar chamber. GO was applied such that it covered either 100% or 20% of its surface by placing GO hydrocolloid drops to the surface to form islands that spread throughout each well. To assess cytotoxicity of GOs, 20 µL of GO hydrocolloids were placed at the bottom of each well of a 96-well microplate and dried for 2 h.

Morphologic features of GO were assessed via TEM imaging using a JEM-1220 microscope (JEOL, Tokyo, Japan) equipped with a Morada 11 megapixel camera (Olympus Soft Imaging Solutions, Münster, Germany) at 80 kV. Samples were prepared by placing a GO droplet onto formvar-coated copper grids (Agar Scientific Ltd., Stansted, UK). Samples were air-dried before they were examined. Zeta potential measurements were carried out using a Nano-ZS90 Zetasizer (Malvern Instruments, Malvern, UK) instrument at 25 °C using the Smoluchowski approximation. Each sample was measured after 120 s of stabilisation at 25 °C.

The surface of GO was analyzed via AFM and SEM. AFM imaging was performed using MFP 3D BioAFM with a commercial triangular cantilever (MPLCT, Bruker, Camarillo, CA, USA), spring constant k = 0.10 N/m in dynamic (AC) mode. SEM imaging was performed using a Quanta 200 microscope (FEI, Hillsboro, OR, USA). Each GO scaffold was pre-dried on the bottom of a 6-well plate in a laminar flow cabinet. Subsequently, each scaffold was contrasted with 1% osmium tetroxide (Sigma-Aldrich, Munich, Germany) and 1% carbohydrazide (Sigma-Aldrich) and dehydrated in increasing concentrations of hexylene glycol (Sigma-Aldrich). Drying was performed using a Polaron CPD 7501 critical point dryer (Quorum Technologies, Laughton, UK).

### 4.2. Cell Culture

Mesenchymal cells were taken from 6-d-old chicken embryos. Fertilized chicken (Ross 308) eggs were purchased from a hatchery and maintained in an incubator under standard conditions. After incubating for 6 days, eggs were removed, and embryos were put on Petri dishes. Using a stereoscopic microscope, different parts of the body, including the hind limb, heart, brain, eye, and blood vessels from the chorioallantoic membrane were dissected and placed into tubes containing trypsin for 24 h at 5–8 °C. Next, trypsin and collected organs were neutralized with DMEM and tissue samples were disintegrated by passing through tips and placed in 75 mL flasks. Cells were maintained in DMEM (Thermo Fisher Scientific, Waltham, MA, USA) supplemented with 10% fetal bovine serum (FBS, Thermo Fisher Scientific) and 1% penicillin/streptomycin (Thermo Fisher Scientific) at 37 °C in a humidified atmosphere of 5% CO_2_ and 95% air in an incubator (NuAire DH AutoFlow CO_2_ Air-Jacketed Incubator, Plymouth, MA, USA). After one day, fresh DMEM was supplied and cells were cultured until a confluence of 80% was reached. Cells were detached using trypsin and placed on experimental 6-well plates (1 × 10^5^ cells per well) and cultured.

HUVEC were obtained from Thermo Fisher Scientific and maintained in the M200PRF basal media supplemented with a large vessel endothelial supplement (Thermo Fisher Scientific) and 1% penicillin/streptomycin (Thermo Fisher Scientific). Cells were maintained at 37 °C in a humidified atmosphere of 5% CO_2_ and 95% air.

### 4.3. Cell Morphology

Primary mesenchymal cells originating from a hind limb (PMC), primary heart cells (PHC), primary neuronal cells originating from the brain (PNC), primary eye cells (PEC), and primary blood vessel cells isolated from the chorioallantoic membrane (PVC) were assessed using an optical inverted microscope (TL-LED, Leica Microsystems GmbH, Wetzlar, Germany) connected to a digital camera (Leica MC190 HD) using LAS V4.10 software. Cells were seeded (1 × 10^5^ cells per well) in 35 mm diameter Petri dishes with and without GO scaffolds. After incubation for 24 h, samples were imaged.

PMC were additionally visualized using a Quanta 200 SEM (FEI, Hillsboro, OR, USA). Cells were seeded (1 × 10^5^ cells per well) in 35 mm diameter Petri dishes with and without GO scaffolds. After a 96-h incubation period, samples were prepared as described by Heckman et al. [46]. Cells were fixed using 2.5% glutaraldehyde in phosphate-buffered saline (PBS) at pH 7.2, contrasted with 1% osmium tetroxide (Sigma-Aldrich) and 1% carbohydrazide (Sigma-Aldrich). Subsequently, cells were dehydrated in increasing concentrations of hexylene glycol (Sigma-Aldrich). Drying was performed using a Polaron CPD 7501 critical point dryer (Quorum Technologies, Laughton, UK).

### 4.4. Cell Viability

The viability of PMC, PHC, PNC, PEC, and PVC grown on the GO scaffold was assessed using a Presto blue assay (Thermo Fisher Scientific). PMC, PHC, PNC, PEC, and PVC were seeded in 96-well microplates with and without GO scaffolds (1 × 10^4^ cells per well in 100 μL of supplemented DMEM media) and incubated for 24 h. Subsequently, 11 μL of the Presto blue reagent were added to each well and results were examined using a Tecan Infinite 200 microplate reader (Tecan, Durham, NC, USA) after a 3 h incubation step in accordance with the manufacturer’s instructions.

### 4.5. Analysis of PMC Migration on a GO Scaffold

To analyze PMC migration on a GO scaffold, cells and GO hydrocolloids were applied to separate wells of a silicone insert (2-well culture inserts, Ibid, Gräfelfing, Germany) attached to a 35 mm dish (µ-Dish 35 mm, high, Ibidi). Initially, 50 μL of GO hydrocolloids (10 μg/mL) were applied to wells of the silicon insert and were allowed to dry using a laminar flow cabinet. Subsequently, PMC (1 × 10^4^ cells per well in a total volume of 70 μL) were seeded in a well without GO. After the cells reached confluence, the silicon insert was gently removed and the cells were incubated for additional 48 h to allow them to migrate to the edge of the GO scaffold. After incubation, media were exchanged for 3 mL DMEM supplemented with4-(2-hydroxyethyl)-1-piperazineethanesulfonic acid (HEPES, final concentration—20 mM). The cells were imaged using a confocal FV-1000 (Olympus Corporation, Tokyo, Japan) microscope equipped with an incubation chamber that maintained temperature of 37 °C. PMC migration on the GO scaffold was analyzed using time-lapse imaging for 18 h (8 min intervals). The analysis of the cell-free area at the initial (0 h) and 18 h timepoint was performed using the MRI Wound Healing Tool macro (http://dev.mri.cnrs.fr/projects/imagejmacros/wiki/Wound_Healing_Tool) for ImageJ software [47]. Migration rate was calculated as the average relative difference between the cell-free areas at 0 and 18 h timepoints using 3 independent experiments. Full time-lapse images of the cells grown on control surfaces and the GO scaffold are available in supplementary information.

### 4.6. mRNA Expression RT-PCR

To determine transcript levels of VEGF-A, FGF2, MyoD1, and PCNA, PMC were cultured in 6-well plates with and without a GO scaffold. GO hydrocolloids were added to a final concentration of 10 μg/mL, and the cells lacking a scaffold were used as a control. Next, 1 × 10^4^ cells per well (passage 3) were seeded onto each well and incubated for 3 days. The cells were washed with PBS, centrifuged and stored at −80 °C. Gene expression levels were measured using the quantitative polymerase chain reaction method (qPCR). PMC were homogenized in the TRIzol Reagent (Thermo Fisher Scientific), and total RNA was extracted according to the manufacturer’s instructions. RNA samples were purified using the SV Total RNA Isolation System (Promega Corporation, Madison, WI, USA) and quantified using a NanoDrop ND 1000 spectrophotometer (Thermo Fisher Scientific). Total RNA (2 mg) was reverse transcribed using a reverse transcriptase with oligo (dT) (Promega) and random primers (TAG Copenhagen A/S, Copenhagen, Denmark). Subsequently, real-time PCR was performed using complementary DNA and gene-specific primer pairs (TAG, Copenhagen A/S, Copenhagen, Denmark) mixed with the LightCycler 480 SYBR Green I Master mix (Roche Applied Science, Penzberg, Germany) in a LightCycler 480 real-time PCR system (Roche Applied Science, Penzberg, Germany). The primers used are listed in Table 1. For each complementary DNA, reactions were performed in triplicate. To determine transcript levels, relative quantification was conducted using the ACTB housekeeping gene.

### 4.7. Quantification of VEGF-A Protein Secretion

PMC cells were seeded (1 × 10^5^) and cultured in 75-mL flasks (covered with GO or not covered as a control) in the supplemented DMEM media. The media were changed after 3 days. After 6 days, all media were removed, and the cells were washed twice with warmed PBS without calcium and magnesium ions (Thermo Fisher Scientific). Subsequently, the cells were starved in DMEM without serum for 2 days, and culture media were centrifuged at 1,500 rpm at 4 °C for 10 min, aliquoted, and stored at −80 °C for later use. Cell-free supernatants of cultures were used for the determination of secreted VEGF-A levels using a chicken VEGF-A kit (Mybiosource, San Diego, CA, USA). The assay was performed according to the manufacturer’s instructions and absorbance was measured using a microplate reader (Tecan Infinite 200, Durham, NC, USA) at 450 nm.

### 4.8. Angiogenesis Potential of PMC

The angiogenic potential of PMCs was analyzed using a HUVEC tube formation assay. Tube formation was performed using μ-Slide Angiogenesis (Ibidi, Gräfelfing, Germany) slides coated with the 10 μL Geltrex Reduced Growth Factor Basement Membrane Matrix (Thermo Fisher Scientific). Cells were suspended in media composed of supplemented M200PRF (80%) and a fresh supplemented DMEM medium (20%) (negative control), supplemented M200PRF (80%) and a post-culture DMEM (20%) obtained as described above for the PMC cultured without the GO scaffold (control group), supplemented M200PRF (80%) and a post-culture DMEM (20%) from the PMC cultured on the GO scaffold (experimental group). HUVEC suspensions (50 μL) were seeded at a density of 1 × 10^4^ cells/well. After 5 h of incubation, HUVEC tubes were fixed with 4% paraformaldehyde (Sigma-Aldrich) for 10 min. Subsequently, tubes were gently washed with PBS (Thermo Fisher Scientific) and stained with the ActinRed 555 ReadyProbes Reagent (Thermo Fisher Scientific). Tubes were imaged using an FV1000 confocal microscope equipped with a 4 X objective (Olympus Corporation). The number of junctions present within the tube mesh was assessed using the ImageJ software [47] and the Angiogenesis Analyzer macro [48].

Proangiogenic properties of culture media used to grow PMC were also verified using an implant taken from the peripheral blood vessel of a chicken embryo. On day 9 of the chicken embryo development, 5 mm samples of a peripheral blood vessel were removed and placed in a 6-well plate with the control medium (post-culture DMEM from the PMC cultured without a GO scaffold) and the experimental medium (post-culture DMEM from the PMC cultured on a GO scaffold). After incubating for 3 days, the vascular implant was fixed with 4% paraformaldehyde (Sigma-Aldrich) for 10 min, stained with hematoxylin–eosin (Sigma-Aldrich), and imaged using an optical inverted microscope (TL-LED, Leica Microsystems GmbH).

### 4.9. Statistical Analysis

The data were analyzed using the one-way analysis of variance (for analysis of three or more groups) or an unpaired *t*-test using GraphPad Prism 8 (GraphPad Software, San Diego, CA, USA). The differences between the groups determined via the one-way analysis of variance were tested with Tukey’s honest significant difference (HSD) post hoc test. The results are shown as means and standard deviations. Differences of *p* < 0.05 were considered significant.

## Figures and Tables

**Table 1:** Primers used for real-time PCR.

Target	Forward Primer	Reverse Primer
FGF2	GGCACTGAAATGTGCAACAG	TCCAGGTCCAGTTTTTGGTC
VEGF-A	TGAGGGCCTAGAATGTGTCC	TCTTTTGACCCTTCCCCTTT
PCNA	TGCACGCATTTGTAGAGACC	AGTCAGCTGGACTGGCTCAT
MyoD1	CGGCGGCTCAGCAAGGTCAAC	CGGCCCGCTGTACTCCATCATG
ATP5B	GTTATTCGGTGTTCGCTGGT	GTAGACCAGAGCGACCTTGG
ACTB	GTCCACCTTCCAGCAGATGT	ATAAAGCCATGCCAATCTCG

## Supplementary Materials

Time-lapse images of the cells grown on control surfaces and the GO scaffold are available in the supplementary information. Supplementary materials can be found at https://www.mdpi.com/1422-0067/21/11/4173/s1.

## Abbreviations

ACTB	actin beta
AFM	atomic force microscope
ATP5B	ATP synthase F1 subunit beta
DMEM	Dulbecco’s Modified Eagle Medium
ECM	extracellular matrix
FGF2	fibroblast growth factor 2
GO	graphene oxide
HH	Hamburger–Hamilton stage
HUVEC	human umbilical vein endothelial cells
MyoD1	myogenic differentiation 1
PCNA	proliferating cell nuclear antigen
PEC	primary eye cells
PHC	primary heart cells
PMC	primary mesenchyme cells
PNC	primary neuronal cells
PVC	primary blood vessel cells
SEM	scanning electron microscope
TEM	transmission electron microscope
VEGF-A	vascular endothelial growth factor A
